# The evaluation of an evidence-based clinical answer format for pediatricians

**DOI:** 10.1186/1471-2431-12-34

**Published:** 2012-03-20

**Authors:** Iva Seto, Michelle Foisy, Brad Arkison, Terry Klassen, Katrina Williams

**Affiliations:** 1 Department of Pediatrics, University of Alberta, Edmonton, Canada; 2 Evaluation and Research Services, University of Alberta, Edmonton, Canada; 3 Manitoba Institute of Child Health, University of Manitoba, Winnipeg, Canada; 4 Department of Developmental Medicine, Royal Children's Hospital, Melbourne, Australia

**Keywords:** Internet, ut [Utilization], Pediatrics, ed [Education], Questionnaires, Delivery of health care, st [Standards], Point-of-Care systems, st [Standards]

## Abstract

**Background:**

Clinicians are increasingly using electronic sources of evidence to support clinical decision-making; however, there are multiple demands on clinician time, and summarised and synthesised evidence is needed. Clinical Answers (CA) have been developed to address this need; the CA is a synthesised evidence-based summary that supports point-of-care clinical decision-making. The aim of this paper is to report on a survey used to test and improve the CA format.

**Methods:**

An online survey was sent to pediatricians via e-mail and posted on a child health clinical standards website. Quantitative data analysis consisted primarily of descriptive statistics; qualitative data analysis consisted of content analysis.

**Results:**

Eighty-three pediatricians responded to the survey. Most respondents found the CA useful or very useful (93%) and agreed or strongly agreed that the layout was effective and allowed them to quickly locate critical information (82%). Quantitative and qualitative data suggested that respondents thought there should be less detail in the linked figures and tables (p = 0.0002), but overall respondents seemed to think there was an appropriate level of detail in most sections of the CA.

**Conclusions:**

Based on the quantitative and qualitative survey responses, major and minor modifications to the CA format were implemented, such as removing forest plots, adding links in each addendum to bring the user back to the front page, and adding an 'Implications for practice' section to the CA. Findings suggest that CAs will be a useful tool for pediatricians; thus, the research team has now begun creating CAs to assist busy clinicians in their day-to-day clinical practice by providing high-quality information for decision-making at the point-of-care.

## Background

The Cochrane Child Health Field is a small knowledge intensive organisation [1] that functions within *The Cochrane Collaboration *to advocate for high-quality scientific evidence that reflects the particular needs of infants, children and youth. Furthermore, the Child Health Field works within the child health community to advocate for decision-making based on finding, understanding, and using the best available evidence. Out of these mandates came the Clinical Answers (CAs) project, which aims to develop short, one-page summaries of evidence to quickly answer questions posed by pediatricians.

Studies suggest that clinicians are increasingly turning to electronic evidence to find information to support clinical decision-making [2-4]. In a study conducted among pediatricians in Ireland, many believed that the quality of patient care depended on the ability of clinicians to use online resources [5]. Among the many online resources available to clinicians are summaries of research evidence to support clinical decision-making, such as Best Bets or UptoDate. Best Bets was originally developed for topics in emergency medicine, and is written by volunteers who register to develop a Best Bet. Evidence is presented in a table, and includes primary studies that are evaluated by authors. UptoDate covers a wide range of topics and is written by physicians; information included spans textbooks to primary studies and is several pages in length. Ketchum *et al *[6] reviewed several of these resources, and found that point-of-care products varied greatly in content, type of evidence, and currency of the evidence. There is a need for consistent and current high-quality evidence delivered in a systematic format; we aim to meet this need by creating and publishing CAs.

Developing short summaries of up-to-date, high-quality evidence is important because clinicians are often unable to keep abreast of current relevant literature [2,4,5,7]. Alper *et al *[8] estimate (from 2005 database figures) that clinicians would require 627.5 hours per month to evaluate all relevant articles - over 20 hours per day, an impossible task. For example, worldwide productivity in asthma research (measured by number of articles published) has doubled from 1994 to 2004 and is continually growing [9,10]. Riordan *et al *[11] found that pediatricians did not have time to track down information and critically appraise evidence while on call; they conclude that pediatricians need easy access to evidence-based answers to common clinical questions.

We chose to develop CAs in the area of respiratory child health, because almost 20% of all reviews published in the Cochrane Database of Systematic Reviews are related to respiratory medicine in children [12]. CAs present evidence from Cochrane systematic reviews, and potentially non-Cochrane systematic reviews, in short one-page electronic summaries with links in text to addendums, that will also be readable in hardcopy. The CA format that we tested consisted of eight separate sections: Question, Answer, Background, Search strategy, Included reviews, Results, Limitations, and References. The addendums included the full search strategy, tables and figures (including forest plots), and references (Additional file 1). Topics for our initial CAs were suggested by pediatricians in the child health field listserv, and also those associated with the Airways and Acute Respiratory Infections Cochrane Review Groups. As CAs are concise, two pediatricians on our team selected the most important outcomes to present for each CA, and decision rules for data extraction and analysis were agreed by our team.

CAs have the potential to be useful for child health clinicians in point-of-care decision-making by providing current high quality evidence in a consistent way to answer common clinical questions. A survey was prepared to test the CA among its intended users. The purpose of the survey was to test the format of the CA, not the particular clinical content of the example used (for example, the survey included questions on the amount and type of information presented, but not whether the clinician agreed with the recommendations for treating bronchiolitis or methodology in producing the CA content). In this paper, we report the survey results and discuss the changes we will implement in the CA format. Conducting this survey assisted us in refining and producing a better product for our future users.

## Method

Potential survey respondents included practicing pediatricians working in diverse clinical and research settings ranging from anaesthesia to oncology. Pediatricians were not randomly sampled; instead, a convenience sample was used. Each survey invitation also had a request for the clinician to send the invitation to colleagues. Our recruitment methods are aimed primarily at European sources as the CAs will be published in *Evidence-Based Child Health*, a journal sponsored by the European Pediatric Association during the project term. Several recruitment methods were employed:

• Email invitations (n = 73) were sent by the Child Health Field to pediatricians in the Field's membership listserv; further invitations were sent by the Cochrane Acute Respiratory Infections Group (n = 46), and Cochrane Airways Group (n = 26) to their contacts.

• An invitation to complete the online survey (including the survey URL) was published in the European Paediatric Association Newsletter, which is sent through e-mail to the Association's membership list.

• A search was conducted through Google Scholar for clinicians who had published in respiratory child health, and where possible, an email invitation (n = 45) to complete the survey was forwarded to these individuals.

• The survey invitation was posted on a European country's national Pediatric Clinical Standards website.

At our University, program evaluation/quality assurance studies (the category the research ethics board assigned us) are not subject to Research Ethics Board review and approval.

### Measures

The online survey was developed by an evaluation research consultant, based on initial input and ongoing review by the research team. The survey addressed several key aspects of the CA format: overall value and efficacy, adequacy of detail provided in sub-sections, suggestions for both format improvements and suggestions for content of future CAs (Additional file 2). An item was also included to determine the Net-Promoter Score (NPS), a tool used to measure customer satisfaction. NPS is derived from a single question that asks respondents to indicate - on an 11-point scale anchored at 0 (not at all likely) and 10 (very likely) - how likely they are to recommend a product to a friend or colleague [13-15]. The NPS is calculated by subtracting the percentage of individuals who are unlikely to recommend a product, referred to as "Detractors" (0-6 on the 11-point scale), from the percentage of individuals who are highly likely to recommend it, called "Promoters" (9-10 on the 11-point scale). A bad recommendation to a colleague is associated with a potential risk to reputation, and as such, most individuals are only likely to recommend a product if they are extremely confident in its quality (Ibid). An NPS that is positive is beneficial in that it represents a potential increase in awareness and use of a product or service.

### Analysis

Quantitative data analysis consisted primarily of descriptive statistics. In addition, for items that asked respondents to comment on the level of detail provided in various sections of the CA, a binomial test was performed to determine whether the proportion of participants who thought there was too much detail (responses of "4" and "5" on the 5-point scale), or too little detail (responses of "1" and "2"), was significantly different from 50%.

A content analysis was performed on the qualitative survey data. For each qualitative question, coding categories were developed by examining participants' responses for recurring patterns. Individual comments were then coded into the appropriate response category by a researcher.

## Results

### Characteristics of survey respondents

Characteristics of our 83 survey respondents are shown in Table 1. On average, respondents had been practicing pediatricians for 17.5 years (SD: 9.78), with the greatest proportion of respondents having practiced between 6-10 and 16-20 years (23% each). Almost all respondents worked in a clinical setting (96%) and over half were engaged in teaching (60%) or research (55%). Forty percent of respondents were involved in clinical care, teaching and research, and 28% were involved in clinical care only. The most common specializations were general pediatrics (28%), hematology/oncology (21%), and pulmonology or respiratory medicine (11%).

**Table 1:** Characteristics of respondents *

Pediatrician's years in practice (N = 83)	N (%)
0 to 5	6 (7.2)

6 to 10	19 (22.9)

11 to 15	15 (18.1)

16 to 20	19 (22.9)

21 to 25	7 (8.4)

26 to 30	9 (10.8)

31+	8 (9.6)

**Setting(s) of work **(N = 83) §	**N (%)**

Clinical care	80 (96.4)

Research	46 (55.4)

Teaching	50 (60.2)

Other †	3 (3.6)

**Specialization **(N = 80)	**N (%)**

Cardiology	0 (0.0)

Critical care medicine	2 (2.5)

Emergency medicine	5 (6.3)

Endocrinology	2 (2.5)

Gastroenterology	0 (0.0)

Hematology-oncology	17 (21.3)

Infectious diseases	6 (7.5)

Nephrology	2 (2.5)

Pulmonology/respiratory	9 (11.3)

Rheumatology	2 (2.5)

Sports medicine	0 (0.0)

Neonatology	3 (3.8)

General pediatrics	22 (27.5)

Community/ambulatory	2 (2.5)

Other ‡	8 (10.0)

* Percentages calculated based on number of respondents for each question

§ Respondents could choose more than one answer to this question

† Administration, quality/effectiveness, and 'unknown'

‡ Dermatology, Pediatric Infectious Diseases and Immunology, Allergy-Immunology, Palliative Care, Neurology, Neurodisability, on call, and training

### Survey responses to questions about the Clinical Answer

Overall, respondents found the CA useful (Table 2). Most respondents found the CA useful or very useful in presenting evidence (93%), agreed or strongly agreed that the CA contained the type of evidence necessary to support clinical decisions (88%) and that they would likely make use of CAs in the future (83%), and indicated that they would likely make use of CAs instead of full-length Cochrane reviews (58%). The positive survey responses were supported by respondents' written feedback: four times more respondents provided feedback that was positive (e.g. "this is excellent - the idea is good and the structure both simple and informative") as opposed to negative (e.g. "lazy way of presenting information").

**Table 2:** Overall usefulness of the Clinical Answer

Question	N (%)				
**N = 82**	**1 (Not at all useful)**	**2**	**3**	**4**	**5 (Very useful)**

How useful do you find this clinical answer format/approach to presenting evidence?	0 (0.0)	1 (1.2)	5 (6.1)	39 (47.6)	37 (45.1)

**Statement**	**N (%)**				

**N = 83**	**1 (Strongly disagree)**	**2**	**3**	**4**	**5 (Strongly agree)**

This clinical answer format/approach contains the type of evidence necessary to support clinical decisions.	0 (0.0)	4 (4.8)	6 (7.2)	40 (48.2)	33 (39.8)

I am likely to make use of these Clinical Answers in the future.	1 (1.2)	4 (4.8)	9 (10.8)	40 (48.2)	29 (34.9)

I would be likely to make use of this Clinical Answer format instead of a Cochrane Review.	5 (6.0)	8 (9.6)	22 (26.5)	32 (38.6)	16 (19.3)

A main purpose of our survey was to identify whether each section of the CA contained an appropriate level of detail (Table 3). Between 65% and 89% of respondents felt there was an appropriate level of detail in each section of the CA, with more than 75% of respondents thinking the amount of detail in the 'Question', 'Answer' and 'Included reviews' sections was appropriate. For respondents who did not think the level of detail in each section was appropriate, a significant number thought there was too much detail in the 'Question' (*p *= 0.04) and 'Linked figures and tables' (*p *= 0.0002) sections and too little detail in the 'Limitations' section (*p *= 0.008).

**Table 3:** Level of detail in each section of the Clinical Answer

How would you describe the level of detail presented in each of the following sections of the Clinical Answer?							
	**N (%)**						
			
**N = 83**	**1 (Too little Detail)**	**2**	**3 (Appropriate level of detail)**	**4**	**5 (Too much detail)**	**Too much detail (%)^**	p-value *

Question	0 (0.0)	1 (1.2)	74 (89.1)	5 (6.0)	3 (3.6)	88.9	0.04 §

Answer	2 (2.4)	7 (8.4)	65 (78.3)	7 (8.4)	2 (2.4)	50.0	1.00

Background	4 (4.8)	9 (10.8)	61 (73.5)	7 (8.4)	2 (2.4)	40.9	0.52

Search strategy	3 (3.6)	6 (7.2)	58 (69.9)	12 (14.5)	4 (4.8)	64.0	0.23

Included reviews	1 (1.2)	7 (8.4)	64 (77.1)	9 (10.8)	2 (2.4)	57.9	0.65

Results	2 (2.4)	9 (10.8)	59 (71.1)	12 (14.5)	1 (1.2)	54.2	0.84

Limitations	3 (3.6)	19 (22.9)	54 (65.1)	5 (6.0)	2 (2.4)	24.1	0.008 †

References	3 (3.6)	12 (14.5)	61 (73.5)	5 (6.0)	2 (2.4)	31.8	0.13

Linked figures and tables	0 (0.0)	2 (2.4)	62 (74.7)	15 (18.1)	4 (4.8)	90.5	0.0002 §

* Calculated based on whether the proportion of participants that felt there was too much detail (responses '4' and '5') as opposed to too little detail (responses '1' and '2') was significantly different from 50%

§A significant number of respondents thought there was too much detail in this section of the Clinical Answer

†A significant number of respondents thought there was too little detail in this section of the Clinical Answer

^of those who scored 1, 2, 4 or 5

Respondents were asked qualitative questions to determine what type of information they felt should be added to or removed from each section of the CA. Most comments related to four recurring themes in the 'Results' section: 1) there should be additional information about the population (location of trials, ages of participants) and intervention (dose and schedule); 2) the word 'significant' should be replaced with the magnitude of the effect; 3) information about heterogeneity should be provided; and 4) statistical terms should be spelled out in full prior to being abbreviated. With the exception of respondents wanting less detail in the search strategy, the qualitative comments overwhelmingly suggested that our CAs should be slightly more detailed. One respondent stated: "I find myself just wanting more information before I view something like this and make a potential change in the way I approach a given clinical situation." Four respondents also shared the opinion that adding "a short concluding remark on what the authors recommend... would be useful."

Regarding questions related to the format of the CA (Table 4), 82% of respondents agreed or strongly agreed that the layout of the CA was an effective way to present content and that it allowed them to quickly locate critical information. Five of nine qualitative comments related to the CA format suggested minor improvements, such as providing links at the end of each section to return to the top of the page. Sixty-five percent of respondents were familiar with the use of GRADE assessments (Grading of Recommendations Assessment, Development and Evaluation), and only 61% of these respondents (40% overall) agreed or strongly agreed that including GRADE assessments in the CA would greatly enhance the quality of the evidence.

**Table 4:** Clinical Answer format

**Please indicate your level of agreement with the following statements about this Clinical Answer format**.					
	**N (%)**				
	
**N = 83**	**1 (Strongly disagree)**	**2**	**3**	**4**	**5 (Strongly agree)**

The table layout of this Clinical Answer is an effective way to present the content.	0 (0.0)	5 (6.0)	10 (12.1)	41 (49.4)	27 (32.5)

This Clinical Answer format allowed me to quickly locate critical information.	0 (0.0)	6 (7.2)	9 (10.8)	41 (49.4)	27 (32.5)

	**N (%)**				
	
N = 54 *	**1 (Strongly disagree)**	**2**	**3**	**4**	**5 (Strongly agree)**

Adding GRADE assessments to this Clinical Answer format would greatly enhance the quality of the evidence presented.	2 (3.7)	1 (1.9)	18 (33.3)	23 (42.6)	10 (18.5)

* All participants were asked if they were familiar with the use of GRADE assessments, and only the 54/83 respondents who answered 'yes' were eligible to answer this question

*GRADE*, Grading of Recommendations Assessment, Development and Evaluation

Lastly, we examined the likelihood of respondents recommending CAs to their colleagues and calculated an NPS of +32.6 from these data (Table 5).

**Table 5:** Net Promoter Score: likelihood of recommending Clinical Answers to colleagues (N = 83)

How likely would you be to recommend Clinical Answers - similar to the example you reviewed - to a colleague?		
**Scale**	**N (%)**	**Aggregate N (%)**

**Detractors**		11 (13.2)
	
0 (very unlikely)	0 (0.0)	
	
1	1 (1.2)	
	
2	0 (0.0)	
	
3	1 (1.2)	
	
4	1 (1.2)	
	
5	0 (0.0)	
	
6	8 (9.64)	

**Passives**		34 (41.0)
	
7	14 (16.9)	
	
8	20 (24.1)	

**Promoters**		38 (45.8)
	
9	15 (18.1)	
	
10 (very likely)	23 (27.7)	
	
**Net Promoter Score (NPS)**: +32.6 *		

* The NPS is calculated by subtracting Detractors (%) from Promoters (%)

### CA format changes

To decide whether to alter the level of detail in any one section of the CA, we created a three-step decision rule. First, we determined the sections in which less than 75% of respondents felt there was an appropriate level of detail (Table 3), and then we carried out a binomial test as described in the Results section. Lastly, we used the qualitative data to determine which types of changes to implement. The binomial test indicated that the amount of detail in the 'Limitations' and 'Linked figures and tables' sections potentially needed to be changed. Upon review of the relevant qualitative comments, we did not find clear direction for increasing the amount of detail in the 'Limitations' section as there were no patterns in the responses; thus we will not be changing this section. For the 'Linked figures and tables' section, the forest plots will be removed because qualitative comments indicated that forest plots are a less familiar presentation format. Furthermore, while the p-value for the 'Question' section was significant, respondents strongly believed there was an adequate amount of detail (89%); therefore this section will not be changed.

Participants also offered a variety of small, easily implemented suggestions that will improve the overall usability of the CA. We will be adding information on geographic location, type of study, age of participants, dose and schedule where possible, spelling out statistical terms in full prior to being abbreviated, and replacing the word 'significant' with the magnitude of the effect. The formatting changes include: adding links to the systematic reviews and trials included in the CAs, and adding a link to each addendum to return to the CA front page.

### GRADE assessments

During development of the CA the team were uncertain whether or not to add GRADE assessments [16] into the format. Because only 65% of respondents knew what GRADE was, and considering the cost in time and resources compared to the benefit of adding GRADE, we decided not to include GRADE in the CA format. A new section will be added: 'Implications for practice.' This section will provide a treatment recommendation and will take into account the overall quality of both the systematic reviews and their included trials for all outcomes of importance.

## Discussion

The findings of our survey suggest that we have developed a useful product for pediatricians that has potential for point-of-care use. Results are positive, and indicate that we need to make only minor changes to the CA format before it is ready for publication. Furthermore, we have a positive NPS score, meaning that in general, clinicians are quite likely to tell their colleagues about CAs. This is of great value in transmitting the news about CAs and facilitating their future use, as Jones *et al *[17] found that medical colleagues are one of the information sources that are perceived to be of great importance to pediatricians' clinical practice.

### The potential benefit of CAs

Attempting to answer all clinical queries as they arise is an important component in evidence-based medicine [5]. In a study conducted in two emergency departments in the US, emergency medicine physicians attempted to answer clinical queries as they arose 81% of the time [18], and were reported to be successful 87% of the time. CAs that address frequently arising clinical questions may increase the chance of success.

Clinicians are often concerned with their ability to keep up to date with the constant flow of new literature relevant to their practice [2,5,7]. Cochrane and other systematic reviews sift through all the available evidence in order to summarize the results of a specific clinical question but they still take substantial time to read, whereas a one-page CA summary could provide a much quicker way to keep up to date.

### Limitations

Because we did not practice random sampling, our survey has a potential for response bias. Those contacted were likely to be more literate in evidence-based medicine because of the sources through which they were contacted, and all respondents reported experience in teaching or research. Furthermore, the CA tested was on respiratory child health, and only a minority of our respondents (11%) specialized in that area. However, we are testing the format and not the content of CAs, and the broad range of child health specialists will help ensure that our CA format is suitable for a wide variety of topics.

With the potential for several thousand participants, we received 83 completed online surveys. However, despite the low response rate, and potential bias from the ratio of respondents from various specializations, we found the survey results to provide coherent, clear information in our product refinement. Furthermore, 21% of respondents were hematology-oncology specialists, and 28% were general pediatricians, which does not reflect the ratio in practice. A possible explanation for this is related to our decision to measure the NPS; a strong Promoter may have sent our survey invitation to a large number of hematology-oncology specialist colleagues.

Lastly, for CAs to be useful we require an increasingly internet and e-resource savvy clinical population [2,3]; however, clinicians also need to be trained in searching and evaluating information for quality and reliability, preferably as early as medical school or the residency stage [19].

## Conclusions

The results of this survey have shown us that the format of the CA, as well as the amount and type of information presented, were well received by evidence users, suggesting if more are developed it will be a useful high quality synthesised and summarised evidence source that can be used at the point-of-care. We have sent the project's final report to the Cochrane Collaboration, and the Cochrane Editorial Unit is currently continuing the work on Clinical Answers. A potential future direction would be to implement the CAs at the point-of-care, and measure the extent of its effect in practice through further surveys or other evaluative method. We aim to build a product that is useful, sustainable, and efficacious for the child health clinical community-- one that meets the critical information needs of busy clinicians in their day-to-day practice decision-making.

## Competing interests

The authors declare that they have no competing interests.

## Authors' contributions

IS managed the project, coordinated the team, recruited participants, carried out initial data analysis, and wrote the introduction and discussion. MF prepared the clinical answer for the survey, carried out further data analysis and wrote the results section. BA prepared the survey and wrote the methods section. TK and KW provided guidance on the project and revised the manuscript. All authors read and approved the final manuscript.

## Pre-publication history

The pre-publication history for this paper can be accessed here:

http://www.biomedcentral.com/1471-2431/12/34/prepub

## Supplementary Material

Additional file 1 **Clinical Answer: Bronchiolitis**. Click here for file

Additional file 2 **Clinical Answer Format: Feedback Survey**. Click here for file

## References

[B1] Makani J Marche S Towards a typology of knowledge-intensive organizations: determinant factors Knowl Man Res Pract 2010 8 265 277 10.1057/kmrp.2010.13

[B2] D'Alessandro DM Lehmann CU, Kim GR, Johnson KB Evidence-Based Medicine and Pediatrics Pediatric Informatics 2009 New York: Springer 133 147

[B3] Davies K Harrison J The information-seeking behaviour of doctors: a review of the evidence Health Info Libr 2007 24 78 94 10.1111/j.1471-1842.2007.00713.x 17584211

[B4] D'Alessandro DM Kreiter CD Peterson MW An evaluation of information-seeking behaviors of general pediatricians Pediatrics 2004 113 t 9 10.1542/peds.113.1.64 14702450

[B5] Prendiville TW Saunders J Fitzsimons J The information-seeking behaviour of paediatricians accessing web-based resources Arch Dis Child 2009 94 633 635 10.1136/adc.2008.149278 19465583

[B6] Ketchum AM Saleh AA Jeong K Type of evidence behind point-of-care clinical information products: a bibliometric analysis J Med Internet Res 2011 13 e21 10.2196/jmir.1539 21335319 PMC3221343

[B7] McCord G Smucker WD Selius BA Hannan S Davidson E Schrop SL Answering questions at the point of care: do residents practice EBM or manage information sources? Acad Med 2007 82 298 303 10.1097/ACM.0b013e3180307fed 17327723

[B8] Alper BS Hand JA Elliott SG Kinkade S Hauan MJ Onion DK How much effort is needed to keep up with the literature relevant for primary care? JMLA 2004 92 429 437 15494758 PMC521514

[B9] Klaewsongkram J Reantragoon R Asthma research performance in Asia-Pacific: a bibliometric analysis by searching PubMed database J Asthma 2009 46 1013 1020 10.3109/02770900903242696 19995139

[B10] Michalopoulos A Falagas ME A bibliometric analysis of global research production in respiratory medicine Chest 2005 128 3993 3998 10.1378/chest.128.6.3993 16354871

[B11] Riordan FA Boyle EM Phillips B Best paediatric evidence; is it accessible and used on-call? Arch Dis Child 2004 89 469 471 10.1136/adc.2003.029413 15102644 PMC1719905

[B12] Bow S Klassen J Chisholm A Tjosvold L Thomson D Klassen T A descriptive analysis of child-relevant systematic reviews in the Cochrane Database of Systematic Reviews BMC Pediatrics 2010 10 34 10.1186/1471-2431-10-34 20487565 PMC2881081

[B13] Reichheld FF The one number you need to grow Harvard Bus Rev 2003 81 46 + 14712543

[B14] Reichheld FF The ultimate question: driving good profits and true growth 2006 Boston: Harvard Business School Press

[B15] Brooks LL Owen R Answering the ultimate question: how Net Promoter can transform your business 2009 San Francisco: Jossey-Bass

[B16] Schunemann HJ Oxman AD Brozek J Glasziou P Jaeschke R Vist GE Grading quality of evidence and strength of recommendations for diagnostic tests and strategies BMJ 2008 336 1106 1110 10.1136/bmj.39500.677199.AE 18483053 PMC2386626

[B17] Jones TH Hanney S Buxton MJ The information sources and journals consulted or read by UK paediatricians to inform their clinical practice and those which they consider important: a questionnaire survey BMC Pediatrics 2007 7 1 10.1186/1471-2431-7-1 17224061 PMC1783849

[B18] Graber MA Randles BD Ely JW Monnahan J Answering clinical questions in the ED Am J Emerg Med 2008 26 144 147 10.1016/j.ajem.2007.03.031 18272092

[B19] Abbas J Schwartz DG Krause R Emergency medical residents' use of Google^® ^for answering clinical questions in the emergency room Proc Am Soc Info Sci Tech 2010 47 1 4

