# Supplementary material for: The evaluation of an evidence-based clinical answer format for pediatricians
Source: BMC Pediatr. 2012 Mar 20;12:34. doi: 10.1186/1471-2431-12-34 (PMC3353169; doi:10.1186/1471-2431-12-34)
Supplement: Additional file 1 — Clinical Answer: Bronchiolitis. [file 1471-2431-12-34-S1.PDF]

EVIDENCE-BASED CHILD HEALTH: A COCHRANE REVIEW JOURNAL
Evid.-Based Child Health (2010)
Published online in Wiley Online Library (onlinelibrary.wiley.com). **DOI:** 10.1002/ebch.111

Evidence-Based Child Health Clinical Answer: Intervention

**QUESTION**	In outpatient and inpatient infants with bronchiolitis, is nebulized hypertonic saline compared to placebo or no treatment effective in reducing rate of hospitalization (outpatients), duration of hospitalization (inpatients) and symptom severity?
**ANSWER**	For inpatients, there is strong, high-quality evidence supporting the use of 3% hypertonic saline to reduce hospital length of stay and clinical severity score. For outpatients, 3% hypertonic saline does not affect length of stay but does significantly reduce clinical severity score.
**BACKGROUND**	• Bronchiolitis is a viral infection that is initially characterized by rhinorrhoea and low-grade fever, which progresses in a few days to cough, respiratory distress, tachypnoea, chest wall retractions, wheeze and/or crackles, and occasionally apnoea.<br>• Bronchiolitis is the most common acute infection of the lower respiratory tract during the first year of life.
**SEARCH STRATEGY**	• **Search strategy**
**INCLUDED REVIEWS**	• The search strategy returned one relevant review that contained seven trials and 581 children. We used an updated (2010) version of this review.<br>• **Table 1** presents characteristics of the included review.
**RESULTS**	• The included review compared 3% hypertonic saline to 0.9% saline. **Table 2** presents a summary of the results.<br>• **Figure 1** and **Figure 2**: 3% hypertonic saline did not affect outpatient rate of hospitalization (RR: 0.63; 95% CI: 0.34, 1.17) but did significantly decrease clinical severity score on days one (MD: -1.28; 95% CI: -1.92, -0.64), two (MD: -2.00; 95% CI: -2.93, -1.07) and three (MD: -2.64; 95% CI: -3.85, -1.43).<br>• **Figure 3**: For inpatients with bronchiolitis, 3% hypertonic saline decreased mean length of stay by 28 hours (MD: -1.16; 95% CI: -1.55, -0.77).<br>• **Figure 4**: For inpatients, hypertonic 3% saline led to a significant decrease in clinical severity score on days one (MD:-0.82; 95% CI: -1.59, -0.06; I^2: 73%), two (MD: -1.14; 95% CI: -1.75, -0.53; I^2: 57%) and three (MD: -1.07; 95% CI: -1.69, -0.44; I^2: 53%).
**LIMITATIONS**	• It is currently unclear which frequency and concentration of hypertonic saline confers the greatest clinical benefit for outpatients and inpatients with bronchiolitis.<br>• The mechanism of action of nebulized hypertonic saline is currently unknown.<br>• Data for inpatient clinical scores must be interpreted with caution, as these studies contained significant heterogeneity (ranging from 53% to 73%).
**REFERENCES**	• **References**

Copyright © 2010 John Wiley & Sons, Ltd.

Search of the Cochrane Database of Systematic Reviews (Issue 11, November 2010)

#1 MeSH descriptor Nebulizers and Vaporizers explode all trees (1670)
#2 MeSH descriptor Saline Solution, Hypertonic explode all trees (294)
#3 (#1 AND #2) (16)
#4 MeSH descriptor Bronchiolitis explode all trees (243)
#5 (#3 AND #4) (1)

References to included reviews

Zhang L, Mendoza-Sassi RA, Wainwright C, Klassen TP. Nebulized hypertonic saline solution for acute bronchiolitis in infants (in press). *Cochrane Database of Systematic Reviews* 2010; in press.

Copyright © 2010 John Wiley & Sons, Ltd.

DOI: 10.1002/ebch.111

Copyright © 2010 John Wiley & Sons, Ltd.

Table I. Characteristics of included reviews

Review title<br>Authors<br>Assessed as up-to-date	Number of studies<br>Study sample size (range)	Population<br>Definition of bronchiolitis	Intervention	Comparison	Outcomes for which data were reported
Nebulized hypertonic saline solution for acute bronchiolitis in infants<br>Zhang L, Mendoza-Sassi RA, Wainwright C, Klassen TP.<br>June 2010	7<br>581 (44-186)	Outpatient and inpatient infants up to two years old.<br>Acute bronchiolitis: first episode of acute wheezing associated with clinical evidence of a viral infection (cough, coryza, or fever). Children with recurrent wheezing were excluded.	Nebulized 3% hypertonic saline.	Nebulized 0.9% saline.	Outpatient: rate of hospitalization, rate of readmission and clinical severity scores.<br>Inpatient: length of hospital stay and clinical severity scores.

Table II. Hospitalization rate, length of stay and clinical severity scores for outpatients and inpatients with bronchiolitis

Population	Outcome	Number of subjects (studies)	Measure of effect (95% CI)	I^2	Quality of evidence (GRADE)[a]
Outpatient	Hospitalization rate	262 (3)	RR: 0.63 (0.34, 1.17)	0%	Low
	Clinical severity score on day 1	65 (1)	MD: -1.28 (-1.92, -0.64)*	--	Low
	Clinical severity score on day 2	65 (1)	MD: -2.00 (-2.93, -1.07)*	--	Low
	Clinical severity score on day 3	65 (1)	MD: -2.64 (-3.85, -1.43)*	--	Low
Inpatient	Length of hospital stay (days)	282 (4)	MD: -1.16 (-1.55, -0.77)*	0%	Moderate
	Clinical severity score on day 1	186 (3)	MD: -0.82 (-1.59, -0.06)*	73%	Low
	Clinical severity score on day 2	186 (3)	MD: -1.14 (-1.75, -0.53)*	57%	Moderate
	Clinical severity score on day 3	156 (3)	MD: -1.07 (-1.69, -0.44)*	53%	Moderate

[a] Quality of evidence was graded as high, moderate, low or very low based on four domains: risk of bias, directness, consistency and precision.
* Significantly favours 3% hypertonic saline. CI: confidence interval; GRADE: Grading or Recommendations, Assessment, Development and Evaluation; MD: mean difference (measured using random effects modelling); RR: risk ratio (measured using random effects modelling).

Evid.-Based Child Health (2010)
DOI: 10.1002/ebch.111

Figure 1. Outpatient hospitalization rate

Study or Subgroup	3% saline Events	3% saline Total	0.9% saline Events	0.9% saline Total	Weight	Risk Ratio M-H, Random, 95% CI
Anil 2010	1	75	1	74	5.0%	0.99 [0.06, 15.48]
Grewal 2009	8	24	13	24	82.4%	0.62 [0.31, 1.21]
Sarrell 2002	2	33	3	32	12.7%	0.65 [0.12, 3.62]
**Total (95% CI)**		**132**		**130**	**100.0%**	**0.63 [0.34, 1.17]**
Total events	11		17			

Heterogeneity: $Tau^2 = 0.00$; $Chi^2 = 0.11$, df = 2 (P = 0.95); $I^2 = 0\%$
Test for overall effect: Z = 1.46 (P = 0.14)

Risk Ratio
M-H, Random, 95% CI

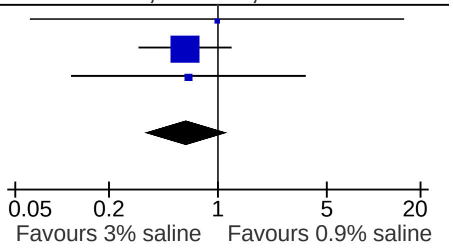

Figure 2. Outpatient clinical severity scores

Study or Subgroup	3% saline Mean	3% saline SD	3% saline Total	0.9% saline Mean	0.9% saline SD	0.9% saline Total	Weight	Mean Difference IV, Random, 95% CI
**2.2.1 Day 1**								
Sarrell 2002	4.36	1.05	33	5.64	1.54	32	100.0%	-1.28 [-1.92, -0.64]
**Subtotal (95% CI)**			**33**			**32**	**100.0%**	**-1.28 [-1.92, -0.64]**
Heterogeneity: Not applicable								
Test for overall effect: Z = 3.90 (P < 0.0001)								
**2.2.2 Day 2**								
Sarrell 2002	2.77	1.4	33	4.77	2.31	32	100.0%	-2.00 [-2.93, -1.07]
**Subtotal (95% CI)**			**33**			**32**	**100.0%**	**-2.00 [-2.93, -1.07]**
Heterogeneity: Not applicable								
Test for overall effect: Z = 4.21 (P < 0.0001)								
**2.2.3 Day 3**								
Sarrell 2002	1.77	2.4	33	4.41	2.57	32	100.0%	-2.64 [-3.85, -1.43]
**Subtotal (95% CI)**			**33**			**32**	**100.0%**	**-2.64 [-3.85, -1.43]**
Heterogeneity: Not applicable								
Test for overall effect: Z = 4.28 (P < 0.0001)								

Mean Difference
IV, Random, 95% CI

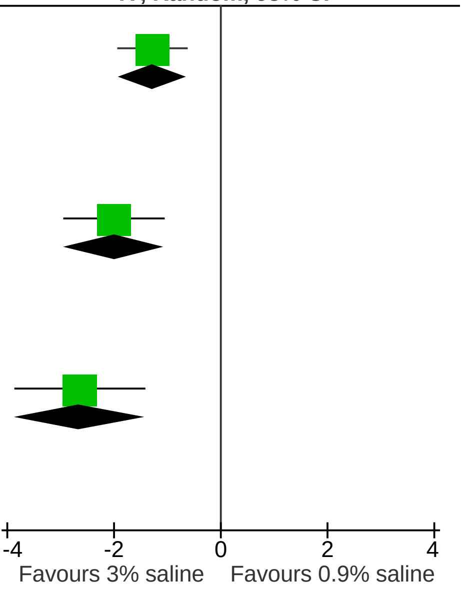

Copyright © 2010 John Wiley & Sons, Ltd.

Evid.-Based Child Health (2010)
DOI: 10.1002/ebch.111

Figure 3. Inpatient length of stay

Study or Subgroup	3% saline Mean	3% saline SD	3% saline Total	0.9% saline Mean	0.9% saline SD	0.9% saline Total	Weight	Mean Difference IV, Random, 95% CI
Kuzik 2007	2.6	1.9	47	3.5	2.9	49	15.7%	-0.90 [-1.88, 0.08]
Luo 2010	6	1.2	50	7.4	1.5	43	48.1%	-1.40 [-1.96, -0.84]
Mandelberg 2003	3	1.2	27	4	1.9	25	19.7%	-1.00 [-1.87, -0.13]
Tal 2006	2.6	1.4	21	3.5	1.7	20	16.4%	-0.90 [-1.86, 0.06]
**Total (95% CI)**			**145**			**137**	**100.0%**	**-1.16 [-1.55, -0.77]**

Heterogeneity: $Tau^2 = 0.00$; $Chi^2 = 1.40$, df = 3 (P = 0.71); $I^2 = 0\%$
Test for overall effect: Z = 5.87 (P < 0.00001)

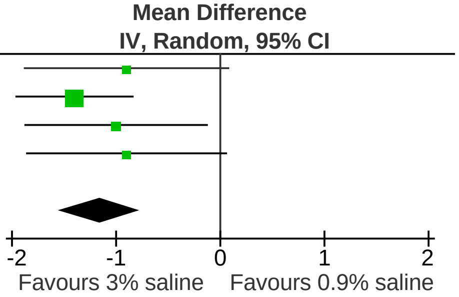

Figure 4. Inpatient clinical severity score

Study or Subgroup	3% saline Mean	3% saline SD	3% saline Total	0.9% saline Mean	0.9% saline SD	0.9% saline Total	Weight	Mean Difference IV, Random, 95% CI
**1.2.1 Day 1**								
Luo 2010	3.4	1.2	50	4.9	1.7	43	35.5%	-1.50 [-2.11, -0.89]
Mandelberg 2003	7.7	1.54	27	7.81	1.49	25	29.9%	-0.11 [-0.93, 0.71]
Tal 2006	6.25	1.1	21	7	1	20	34.6%	-0.75 [-1.39, -0.11]
**Subtotal (95% CI)**			**98**			**88**	**100.0%**	**-0.82 [-1.59, -0.06]**
Heterogeneity: $Tau^2 = 0.33$; $Chi^2 = 7.49$, df = 2 (P = 0.02); $I^2 = 73\%$								
Test for overall effect: Z = 2.11 (P = 0.03)								
**1.2.2 Day 2**								
Luo 2010	2.2	1.1	50	3.8	1.5	43	39.9%	-1.60 [-2.14, -1.06]
Mandelberg 2003	6.41	1.4	24	6.92	1.62	25	27.5%	-0.51 [-1.36, 0.34]
Tal 2006	5.35	1.3	21	6.45	1	20	32.6%	-1.10 [-1.81, -0.39]
**Subtotal (95% CI)**			**95**			**88**	**100.0%**	**-1.14 [-1.75, -0.53]**
Heterogeneity: $Tau^2 = 0.17$; $Chi^2 = 4.70$, df = 2 (P = 0.10); $I^2 = 57\%$								
Test for overall effect: Z = 3.65 (P = 0.0003)								
**1.2.3 Day 3**								
Luo 2010	1.5	0.5	45	2.9	0.7	40	54.4%	-1.40 [-1.66, -1.14]
Mandelberg 2003	5.81	1.68	21	6.08	2.03	23	21.0%	-0.27 [-1.37, 0.83]
Tal 2006	4.7	1.5	13	5.72	1	14	24.6%	-1.02 [-1.99, -0.05]
**Subtotal (95% CI)**			**79**			**77**	**100.0%**	**-1.07 [-1.69, -0.44]**
Heterogeneity: $Tau^2 = 0.17$; $Chi^2 = 4.24$, df = 2 (P = 0.12); $I^2 = 53\%$								
Test for overall effect: Z = 3.36 (P = 0.0008)								

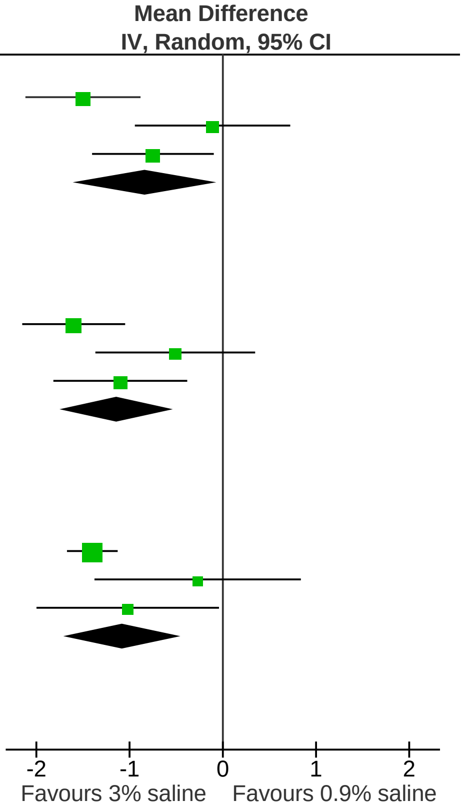

Copyright © 2010 John Wiley & Sons, Ltd.

Evid.-Based Child Health (2010)
DOI: 10.1002/ebch.111
