# Supplementary material for: The evaluation of an evidence-based clinical answer format for pediatricians
Source: BMC Pediatr. 2012 Mar 20;12:34. doi: 10.1186/1471-2431-12-34 (PMC3353169; doi:10.1186/1471-2431-12-34)
Supplement: Additional file 2 — Clinical Answer Format: Feedback Survey. [file 1471-2431-12-34-S2.DOC]

# Clinical Answer Format: Feedback Survey

| 1. **For how many years have you been a practicing pediatrician?** | |
| --- | --- |
|  | Years |

| 1. **In which of following settings do you work (please select all that apply)?** | |
| --- | --- |
|  | Clinical care |
|  | Research |
|  | Teaching |
|  | Other (please specify) |

| 1. What is your area of speciality within pediatrics? | |
| --- | --- |
|  | Cardiology |
|  | Critical Care Medicine |
|  | Emergency Medicine |
|  | Endocrinology |
|  | Gastroenterology |
|  | Hematology-Oncology |
|  | Infectious Diseases |
|  | Nephrology |
|  | Pulmonology/Respiratory |
|  | Rheumatology |
|  | Sports Medicine |
|  | Neonatology |
|  | General Paediatrics |
|  | Community/Ambulatory |
|  | Other (Please specify) |

| 1. **Overall, how useful do you find this Clinical Answer format/approach to presenting evidence?** | | | | |
| --- | --- | --- | --- | --- |
| Not at all useful |  |  |  | Very useful |
| 1 | 2 | 3 | 4 | 5 |

| 1. **This Clinical Answer format/approach contains the type of evidence necessary to support clinical decisions.** | | | | |
| --- | --- | --- | --- | --- |
| Strongly disagree |  |  |  | Strongly agree |
| 1 | 2 | 3 | 4 | 5 |

Skip if question 5 is greater than “3”

| 1. **Why do you feel the format/approach of this Clinical Answer does not contain the evidence necessary to support clinical decisions, and how could the format be improved in this regard?** |
| --- |
|  |

| 1. **How would you describe the level of detail presented in each of the following sections of the Clinical Answer?** | | | | | |
| --- | --- | --- | --- | --- | --- |
|  | **Too little**  **detail** |  | **Appropriate level of detail** |  | **Too much**  **detail** |
| 1. Question | 1 | 2 | 3 | 4 | 5 |
| 1. Answer | 1 | 2 | 3 | 4 | 5 |
| 1. Background | 1 | 2 | 3 | 4 | 5 |
| 1. Search strategy | 1 | 2 | 3 | 4 | 5 |
| 1. Included reviews | 1 | 2 | 3 | 4 | 5 |
| 1. Results | 1 | 2 | 3 | 4 | 5 |
| 1. Limitations | 1 | 2 | 3 | 4 | 5 |
| 1. References | 1 | 2 | 3 | 4 | 5 |
| 1. Linked figures and tables | 1 | 2 | 3 | 4 | 5 |

Skip if question 7a – 7i is greater than/equal to “Appropriate level of detail”

| 1. **What additional information should be included in this Clinical Answer format?** |
| --- |
|  |

Skip if question 7a – 7i is less than/equal to “Appropriate level of detail”

| 1. **What information would you exclude from this Clinical Answer format?** | | | | | |
| --- | --- | --- | --- | --- | --- |
|  | | | | | |
| 1. **Please indicate your level of agreement with the following statements about this Clinical Answer format.** | | | | | |
|  | **Strongly disagree** |  |  |  | **Strongly agree** |
| 1. The table layout of this Clinical Answer is an effective way to present the content. | 1 | 2 | 3 | 4 | 5 |
| 1. This Clinical Answer format allowed me to quickly locate critical information. | 1 | 2 | 3 | 4 | 5 |
| 1. I am likely to make use of these Clinical Answers in the future. | 1 | 2 | 3 | 4 | 5 |
| 1. I would be likely to make use of this Clinical Answer format instead of a Cochrane Review. | 1 | 2 | 3 | 4 | 5 |

| 1. **Are you familiar with the use of GRADE assessments (i.e., a score that indicates the overall quality of synthesized evidence)?** | |
| --- | --- |
|  | Yes |
|  | No |

Skip if question 11 equals “no”

| 1. **Adding GRADE assessments to this Clinical Answer format would greatly enhance the quality of the evidence presented.** | | | | |
| --- | --- | --- | --- | --- |
| Strongly disagree |  |  |  | Strongly agree |
| 1 | 2 | 3 | 4 | 5 |

| 1. **How likely would you be to recommend Clinical Answers – similar to the example you reviewed – to a colleague?** | | | | | | | | | | |
| --- | --- | --- | --- | --- | --- | --- | --- | --- | --- | --- |
| Very unlikely |  |  |  |  |  |  |  |  |  | Very likely |
| 0 | 1 | 2 | 3 | 4 | 5 | 6 | 7 | 8 | 9 | 10 |

| **14. We are producing 25 Clinical Answers in the area of respiratory medicine. What other PICO-style questions (population, intervention, comparison, and outcome) would you like to be addressed with a Clinical Answer? Please specify the type of information you would like (e.g., diagnosis, prognosis or treatment) and the topics that interest you (e.g., asthma, cystic fibrosis, croup).** |
| --- |
|  |

| 1. **Do you have any additional comments about the Clinical Answer format, or specific suggestions for how the format can be improved?** |
| --- |
|  |
